# From *Klebsiella* and *Candida* to artificial intelligence: a perspective from infectious diseases doctors and researchers

**DOI:** 10.3389/fmed.2025.1676920

**Published:** 2025-09-29

**Authors:** Daniele Roberto Giacobbe, Cristina Marelli, Marco Muccio, Sabrina Guastavino, Ylenia Murgia, Sara Mora, Alessio Signori, Nicola Rosso, Antonio Vena, Mauro Giacomini, Cristina Campi, Michele Piana, Matteo Bassetti

**Affiliations:** ^1^UO Clinica Malattie Infettive, IRCCS Ospedale Policlinico San Martino, Genoa, Italy; ^2^Department of Health Sciences (DISSAL), University of Genoa, Genoa, Italy; ^3^Oncostat, CESP, Inserm U1018, Université Paris-Saclay, Labeled Ligue Contre le Cancer, Gustave Roussy, Villejuif, France; ^4^Institut Curie - INSERM U1331, Team Statistics Applied to Personalized Medicine, Paris, France; ^5^Department of Mathematics (DIMA), University of Genoa, Genoa, Italy; ^6^Department of Informatics, Bioengineering, Robotics and System Engineering (DIBRIS), University of Genoa, Genoa, Italy; ^7^UO Information and Communication Technologies, IRCCS Ospedale Policlinico San Martino, Genoa, Italy; ^8^Section of Biostatistics, Department of Health Sciences (DISSAL), University of Genoa, Genoa, Italy; ^9^IRCCS Ospedale Policlinico San Martino, Genoa, Italy; ^10^Life Science Computational Laboratory (LISCOMP), IRCCS Ospedale Policlinico San Martino, Genoa, Italy

**Keywords:** artificial intelligence, machine learning, prediction, invasive candidiasis, carbapenem resistance

## Abstract

The advent of artificial intelligence (AI) and machine learning (ML) is progressively influencing clinical reasoning in infectious diseases, particularly in the management of septic shock where timely empirical antimicrobial therapy is crucial. In this perspective, we discuss how AI and ML approaches intersect with established clinical decision-making processes through two examples from our research and practice: prediction of bloodstream infection by carbapenem-resistant *Klebsiella pneumoniae* and prediction of candidemia. Traditionally, risk estimation has relied on interpretable models such as logistic regression, offering clinicians transparent insights into the contribution of specific risk factors. In contrast, some ML models leverage complex relationships within large datasets. Despite expectations, in several cases these complex models have not consistently outperformed classical approaches yet, a phenomenon we refer to as the “accuracy paradox,” possibly stemming from limitations in data specificity and granularity. Furthermore, the opacity of many ML models still challenges their integration into clinical practice, raising ethical and practical concerns around explainability and trust. While explainable AI offers partial solutions, ML may also capture hidden patterns undetectable through classical reasoning that could be unexplainable to clinicians *per definition*. Achieving a reasonable and shared balance will require continued collaboration between clinicians, data scientists, and ethicists. As the field evolves, future research should prioritize the development of models that not only perform well but can also integrate meaningfully into the complex cognitive processes underpinning bedside clinical reasoning.

## Introduction

As infectious disease specialists, part of our routine clinical work includes consultations for patients with septic shock across various hospital wards. In these patients, empirical antimicrobial therapy is typically initiated while awaiting blood culture results for etiological diagnosis, which may take up to 48–72 h. This practice reflects the deleterious prognostic impact of delaying effective therapy (1–5).

At the bedside, two crucial therapeutic considerations often arise in patients with septic shock in our hospital: (i) whether empirical antibacterial therapy should include coverage against carbapenem-resistant Gram-negative bacteria, and (ii) whether empirical antifungal therapy should be added in the suspicion of candidemia. Over recent decades, these considerations have become core to our clinical research interests (6–8). As a result, clinical reasoning and research interpretation have become increasingly intertwined, mutually enhancing and deepening our approaches to both fields.

Recently, the terms artificial intelligence (AI), referring to machines capable of performing human-like tasks, and machine learning (ML), a subset of AI referring to machines capable of learning a specific task from data without being programmed to do so, have gained prominence in the infection prediction literature, inevitably intersecting with our key areas of clinical practice and research (9–16). Importantly, this shift is not merely terminological; the principles underlying AI and ML raise new considerations, reshaping our clinical and research reasoning.

With the support of expert statisticians, mathematicians, and bioengineers, while maintaining a clinically oriented perspective, this brief perspective discusses how AI and ML are beginning to reshape our field. We illustrate these changes through two longstanding topics in our research and clinical practice that guide empirical therapeutic decisions in septic shock: prediction of bloodstream infection (BSI) by carbapenem-resistant *Klebsiella pneumoniae* (CRKP) and prediction of candidemia (i.e., BSI by *Candida spp.*).

## Clinical reasoning at the bedside for suspected CRKP BSI

When selecting empirical therapy for patients with severe acute conditions (e.g., septic shock) and suspected BSI, we carefully weigh the risk of CRKP etiology. If this risk is deemed negligible, empirical therapy without CRKP coverage is prescribed. Conversely, if the risk is appreciable, empirical therapy with CRKP coverage is initiated, pending blood culture results to adjust treatment accordingly (e.g., de-escalation to non-CRKP-covering regimens if other non-resistant organisms and/or carbapenem-susceptible *Klebsiella pneumoniae* are identified).

One might wonder whether it would be preferable to routinely start with CRKP-active regimens and de-escalate after blood cultures. While theoretically justifiable in consistently high-risk settings, such scenarios are rare. Routine use of broad CRKP coverage is usually unjustifiable from an antimicrobial stewardship perspective, as it fosters resistance development without clear survival benefit (17–23). Hence, accurately estimating CRKP risk is central to guiding empirical therapy.

## CRKP BSI risk prediction from classical models

Estimating CRKP risk highlights the connection between clinical practice and research. Risk estimation in real world practice relies on identifying risk factors derived from research studies, typically using logistic regression or other classical statistical models (24–27).

In univariable logistic regression, the association between a potential risk factor (e.g., prior hospitalization) and the event of interest (e.g., CRKP BSI) is quantified. In this example, prior hospitalization is the independent variable (x₁) and CRKP BSI is the dependent variable (y). A dataset comprising x₁ and y values from multiple previous patients is used to “train” the logistic regression model to predict y based on x₁. More in detail, training on the actual data in the dataset means logistic regression coefficients (intercept *β*₀ and slope β₁) are calculated through maximum likelihood estimation (28). Then, the probability (*p*) of CRKP BSI for any new patient based on the presence or not of previous hospitalization can be calculated through the following formula:


$$p=p(x_{1})=P\;(y=1|x_{1})=\sigma (\beta_{0}+\beta_{1}x_{1})=\frac{1}{1+e^{-(\beta_{0}+\beta_{1}x_{1})}}$$

A linearized version via the logit function clarifies interpretation:


$$\text{logit}(p)=\log(\frac{p}{1-p})=\beta_{0}+\beta_{1}x_{1}$$

Indeed, exponentiating *β*₁ yields the odds ratio (OR) for x₁, interpreted as the odds of CRKP BSI in patients with vs. without prior hospitalization. Notably, although OR reflects association rather than causation, it provides clinicians with a quantifiable sense of risk.

Risk scores typically incorporate multiple variables and are derived not from univariable but from multivariable models (e.g., multivariable logistic regression) (28), with *p* depending on multiple independent variables. Consequently, multivariable logistic regression includes several independent variables ( $x_{1},..,x_{n}$ ), each with its coefficient ( $\beta_{1},\ldots ,\beta_{n}$ ), according to the following formula (linearized via the logit function):


$$\text{logit}(p)=\beta_{0}+\sum_{i=1}^{n}\beta_{i}x_{i}$$

For example, the Giannella score predicts CRKP BSI in colonized patients, after training of a multivariable logistic regression model led to the identification of predictive factors such as ICU admission (OR 1.65), abdominal procedures (OR 1.87), chemotherapy/radiation (OR 3.07), and additional colonization sites (OR 3.37 per site) (25). Such a classic approach of predicting CRKP BSI via logistic regression and presenting the results via OR is certainly not obsolete, as several similar studies from different parts of the world have been published in the last 12 months (29–34), with the persistence of this technique’s application over the years testifying to its usefulness for both researchers and clinicians.

Of note, *β* coefficients are also called parameters or weights in ML terminology (we will continue using the terms β coefficients throughout the manuscript for consistency).

## The advent of AI and ML in risk prediction: what is truly new?

Recent literature abounds with studies using ML models to predict severe infections (9–12, 14–16). A source of possible confusion for clinicians is that logistic regression is frequently included among ML techniques in these studies, and also introductory ML courses often begin with describing logistic regression as a ML model. Technically, this is valid: logistic regression models learn from prior x = ( $x_{1},..,x_{n}$ ) and y data to predict outcomes for new patients, meeting ML’s definition (35).

Where, then, lies the difference between traditional logistic regression and the recent surge in ML-based predictions? One might assume that novel, more accurate models have emerged only recently, justifying the influx of studies. However, this is incorrect. ML models like neural networks (NNs) date back to the mid-20th century (36). What was truly lacking until a very few years ago was the computational power and data volume (“big data”) necessary to train complex ML models in a reasonable amount of time and without overfitting (i.e., when a model learns the training data too well, possibly including its noise and outliers, leading to poor performance on new, unseen data (37)).

## The example of neural networks and deep learning

**Figure 1:**
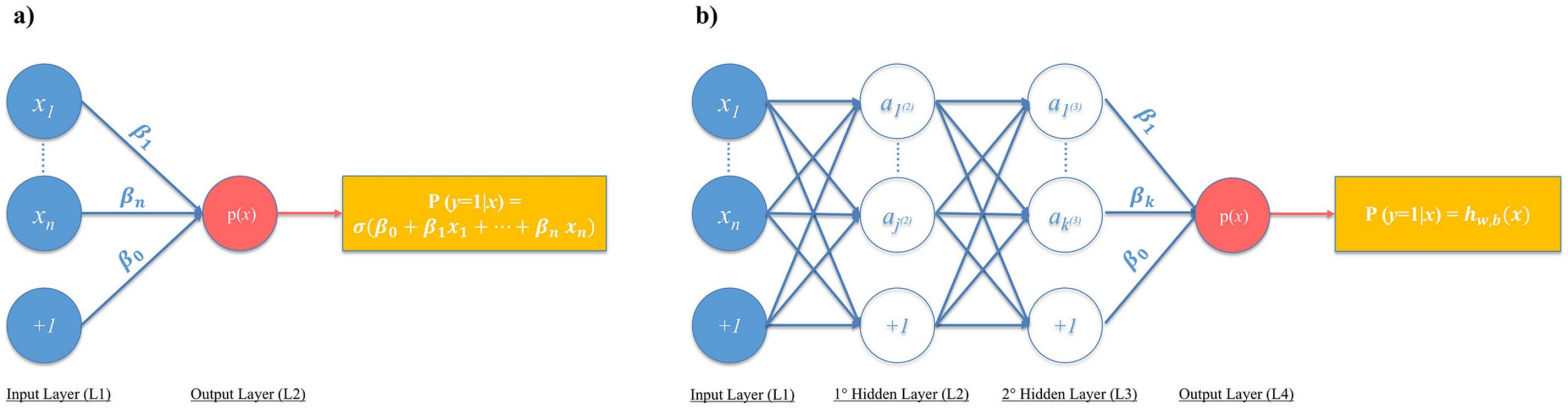
Comparison between: **(a)** multivariable logistic regression (2 layers), **(b)** neural network (just 2 hidden layers for concept illustration purposes). *x*_1_… *x*_n_: Input variables (features); +1: Bias node; *β*_0_: Logistic regression intercept; *β*_1_… *β*_n_: Logistic regression coefficients (weights); p(*x*) = P(*y* = 1∣*x*): Predicted probability; *x*: Vector of input variables; *σ*: Sigmoid function; *α*_1_^(2)^… *α*_j_^(2)^: 1^st^ hidden layer transformed features; *α*_1_^(3)^… *α*_k_^(3)^: 2^nd^ hidden layer transformed features; *β*_o_… *β*_k_: NN final weights; *h*_w,b_: NN prediction function (in which w represent the synaptic connection weights between layers, and b the bias terms). Figure created using Microsoft PowerPoint (Office 2016, Microsoft Corporation, Redmond, WA, USA).

With modern computational advances and data availability, it may be expected that more complex ML models could deliver more accurate risk predictions than classical models (e.g., logistic regression). For instance, NNs capture intricate feature interactions by combining them through intermediate layers. Conceptually, logistic regression resembles a two-layer NN (input and output), whereas NNs include at least one “hidden” layer (Figure 1). Multiple hidden layers constitute “deep learning,” reflecting the depth of learned associations (38).

Unlike logistic regression’s two types of coefficients (β_0_ and β_1…n_, with n equal to the number of features), NNs involve other numerous additional trainable coefficients across hidden layers (Figure 1). Although architectures vary across complex ML models other than NNs, their shared principle is capturing complex patterns through training of multiple additional *β* coefficients potentially enhancing predictive accuracy.

If sufficient high-quality data are available to prevent overfitting, complex models should theoretically outperform simpler ones (e.g., logistic regression) in predicting infections and resistance, informing antimicrobial choices in septic shock. Yet, the reality is more nuanced, as discussed below.

## The accuracy paradox

Accuracy measures a model’s ability to correctly classify true positives (TP) and true negatives (TN) (39):


$$\text{Accuracy}=\frac{\mathrm{TP}+\mathrm{TN}}{N}$$

Where N is the total number of observations, i.e., the sum of TP, TN, false negatives, and false positives.

**Figure 2:**
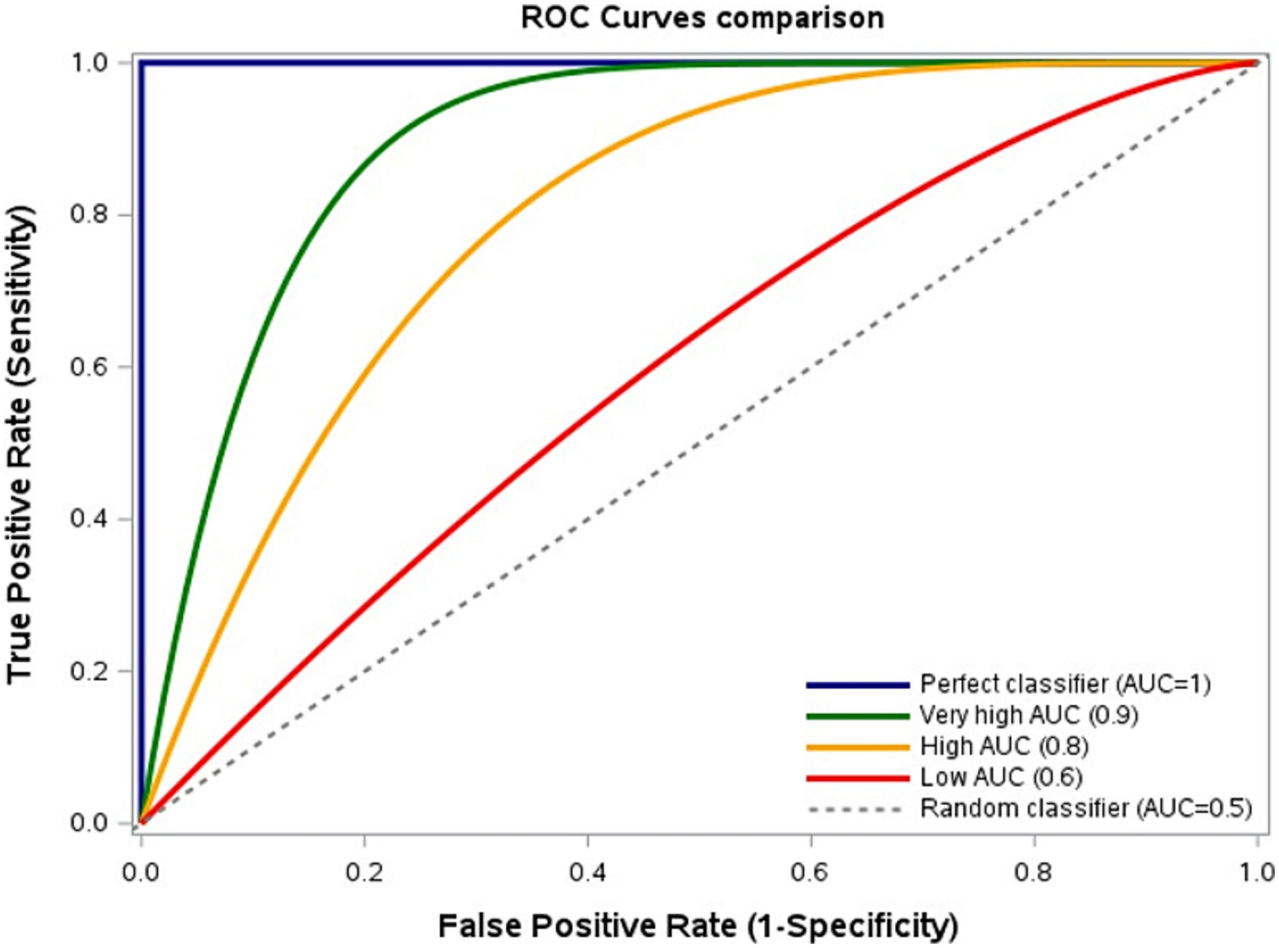
Model discrimination based on sensitivity-specificity trade-offs. Receiver Operating Characteristic (ROC) curves comparing True Positive Rate (TPR, sensitivity) and False Positive Rate (FPR, 1–specificity) across thresholds. Area Under the Curve (AUC): Highest = 1, Very high = 0.9, High = 0.8, Low = 0.6, Lowest = 0.5. Curves nearer the upper-left corner indicate better discrimination. Figure created using SAS software (version 9.4, SAS Institute Inc., Cary, NC, USA).

In this regard, it is of nonetheless of note that another performance measure is commonly used to assess the model discriminatory ability for classification problems (due to technical reasons that are outside the scope of the present paper), the area under the curve (AUC). The AUC evaluates the model discriminatory ability across all thresholds, reflecting sensitivity-specificity trade-offs (39). Higher AUC indicates superior discrimination between positive and negative cases (Figure 2).

Recent ML infection prediction studies trained on very large samples of thousands of patients frequently report AUCs ≥0.6–0.7 (11, 40, 41). However, we encourage scrutiny also of other performance metrics (e.g., sensitivity, specificity). Indeed, this may allow to note more intuitively that, despite training complex models on extensive datasets, their sensitivity and specificity often do not surpass—and sometimes underperform—those of classical models trained on smaller datasets (11, 24–27, 40, 41). Possible explanations vary (e.g., selection biases, suboptimal architectures). A key factor may nonetheless be features specificity and granularity: large datasets often rely on administrative codes and routine, nonspecific laboratory results, whereas smaller datasets capture granular clinical details (e.g., invasive devices, acute conditions). Since manual collection of granular features from thousands to millions health records, as required not to overfit complex ML predictive models, is frequently unfeasible, current limitations in automated granular feature extraction may constrain ML’s potential (42, 43).

We term this the “accuracy paradox” (technically, it should be the “AUC paradox,” but we think using the term “accuracy” could be more intuitive for readers), i.e., the apparent lack of performance improvement despite leveraging complex models and big data. However, advancements in AI-driven feature extraction from electronic health records (including unstructured text) may soon resolve this paradox (44, 45). This leads us to another crucial consideration: explainability.

## Explainable artificial intelligence (XAI)

Figure 1 illustrates another key point. In logistic regression, exponentiating *β* coefficients yields interpretable ORs. In contrast, NNs’ hidden layers usually obscure feature contributions, rendering them “black-box” models (46). This opacity extends to other complex ML techniques and challenges their clinical adoption. Indeed, interpretability underpins clinical reasoning. Recognizing independent risk factors, although reflecting association and not causation, yet supports plausible causality (e.g., the association of prior rectal CRKP colonization with the possible development of CRKP BSI is plausible from a pathophysiological standpoint, reflecting possible translocation into the bloodstream). This also means that, if researchers and clinicians encounter “strange” or absent associations (in terms of their plausible causality), this may help them to recognize possible biases or data errors, issues harder to detect with black-box models.

XAI seeks to approximate black-box predictions through more interpretable (but expectedly less accurate) models, aimed to identify influential features (47–49). Yet, such explanations usually carry inherent uncertainty and remain less robust than those from inherently interpretable models (46, 50–52). This also means that if a simpler, interpretable model matches a black-box model’s accuracy, the former is preferable. Moreover, explainability aligns with the autonomy principle in biomedical ethics, facilitating shared decision-making (53). XAI’s evolution aims to enhance explanation reliability to favor this crucial alignment. Still, further considerations arise, as discussed next.

## Should we always explain? Insights from candidemia research

As discussed above, we suspect suboptimal performance of some complex models stems from reliance on nonspecific data. However, the modest but present predictive power achievable from such data warrants additional reflection. To illustrate our point, we present an example from research on the use of ML to predict candidemia, an emerging field of research with a handful of original studies published in recent years (11, 13, 14, 16, 54, 55). In the largest of these studies at the time of this perspective, a deep learning model trained on nonspecific laboratory results (e.g., platelets, serum creatinine) and previous *Candida* colonization achieved sensitivity 70%, specificity 58%, positive predictive value 16%, and negative predictive value 95% for distinguishing candidemia from bacteremia across >12,000 BSI events (11). These metrics mirror those from classical models using known risk factors, suggesting ML’s capacity to leverage complex feature interactions imperceptible to clinicians (none of us, as clinicians, is indeed able to predict candidemia based only on platelet count and other nonspecific laboratory features) (56, 57). Notably, integrating nonspecific features with established biomarkers of candidemia (e.g., serum *β*-D-glucan) yielded no additive predictive benefit in our experience, highlighting the need for further research (11). Should future methods successfully harness such interactions of nonspecific features to improve predictions, clinical reasoning paradigms may shift. We might need to accept that part of our predictions cannot be meaningfully explained—even with XAI. This raises important ethical questions. Indeed, while improved predictions may benefit patients, opaque reasoning challenges autonomy and informed consent. Finding any possible acceptable compromise will require robust multidisciplinary dialogue.

## Discussion

In this perspective, we have reflected on how the growing integration of AI and ML into infection prediction is beginning to reshape the clinical reasoning traditionally applied to empirical antimicrobial therapy in critically ill patients. Through the examples of CRKP BSI and candidemia, we highlighted how these emerging tools challenge established paradigms rooted in classical risk scores.

Classical statistical models, such as logistic regression, have long been appreciated for their transparency and alignment with clinicians’ intuitive reasoning processes. They provide interpretable outputs, such as ORs, which allow healthcare professionals to understand and validate the rationale behind risk predictions. These characteristics have facilitated their integration into both research and clinical practice.

In contrast, modern ML models may (albeit not always) offer the potential for enhanced predictive accuracy through their capacity to model complex, non-linear relationships within large datasets. As we have discussed, this potential has not always translated into clear improvements in real-world predictive performance. This “accuracy paradox” may stem in part from the nature of the available data: large datasets often lack the clinical granularity of smaller, manually curated cohorts, relying instead on administrative codes and routine laboratory values with limited direct relevance to the pathophysiology of infection. On the other hand, it should be noted that the literature also includes positive examples of ML-enhanced improved prediction of antimicrobial resistance using limited electronic health record and laboratory data. Yang and colleagues reported on an improved performance of ML models over classical logistic regression approaches for predicting resistance to four first-line antibiotics in patients with complicated urinary tract infections (58). In addition, Vihta and colleagues exploited historical antimicrobial resistance and antibiotic use data to predict through ML techniques future resistance in different hospitals in England, showing the models ability to capture complex relationships across use of different antibiotics and different types of resistance to predict future resistance (59). While any generalizability of these proof of concept findings requires external validation across diverse clinical settings (this may require additional efforts in low- and middle-income countries, where a more limited deployment of digital infrastructure and electronic health records could delay external validation and consequently compromise the local applicability of predictive models), the presence of positive examples underscores the possibility to effectively circumvent the accuracy paradox, which requires a complex but achievable balance in terms of dataset size, feature selection, performance metrics, and generalizability to other clinical settings.

Beyond accuracy, a further critical issue is explainability. Complex ML models frequently operate as black boxes, obscuring the pathways by which predictions are generated. This opacity limits clinicians’ ability to scrutinize predictions for plausibility, undermines trust, and complicates shared decision-making with patients. The field of XAI has emerged to address these limitations, but current approaches remain imperfect and less robust than explanations derived from traditional models. The preliminary experience with candidemia prediction nonetheless underscores a further nuance: models can capture subtle patterns from nonspecific features, achieving predictive performance comparable to classical risk scores. While this hints at untapped opportunities to enhance clinical decision-making, it also raises fundamental questions about the acceptability of predictions that cannot be meaningfully explained in clinical terms. In this regard, it should be noted that some authors have suggested that the critique of AI models as black boxes is controversial, since clinicians reasoning also operates within equally opaque decision-making processes that cannot be fully described or reproduced, but which lead expert clinicians to integrate, not necessarily awarely, subtle cues that ultimately produce effective outcomes (60). This can be used to support the argument that it is not the nature of the reasoning (human vs. machine) and the explanation of predictions that are important, but rather whether the predictions are correct, useful, safe, and appropriately validated. However, in our view, this argument reflects an incomplete picture, at least from two standpoints. The first is that of abductive reasoning, i.e., one could assume, as educated guess, that a clinician’s opaque reasoning and intuitions are similar to those of other clinicians (since both the former and the latter are humans and share evolutionary patterns of reasoning processes), but the same may not be true for machines. To illustrate our point with an example, we cannot rule out the possibility that, even if predictions based on optimized and validated ML models may be more accurate than human ones, when errors occur, these could be easily identified and prevented by expert clinicians thanks to shared human intuition or classical human reasoning. Certainly, this would require confirmation through rigorous research on the human ability to anticipate or rapidly recognize ML-based prediction errors before they become actionable; in the meantime, we nonetheless believe this possibility already and further underscores the need for AI as an assistant rather than a substitute for clinicians. The second standpoint, as partly anticipated above, is that of shared decision-making between clinicians and patients, which could be hampered by clinicians’ inherently limited or absent ability to explain ML-assisted decisions when they derive from black-box models, and by patients’ consequent inability to truly understand the related informed consent, also regarding data use and sharing for research purposes. In this light, achieving a reasonable and shared balance will require continued collaboration between clinicians, data scientists, and ethicists. As the field evolves, future research should prioritize the development of models that not only perform well but can also integrate meaningfully into the complex cognitive processes underpinning bedside clinical reasoning.

## Data Availability

The original contributions presented in the study are included in the article/supplementary material, further inquiries can be directed to the corresponding author.

## References

[1] Deresinski S. Principles of antibiotic therapy in severe infections: optimizing the therapeutic approach by use of laboratory and clinical data. Clin Infect Dis. (2007) 45:S177–83. doi: 10.1086/519472, PMID: 17712744

[2] Baltas I Stockdale T Tausan M Kashif A Anwar J Anvar J. Impact of antibiotic timing on mortality from gram-negative bacteraemia in an English district general hospital: the importance of getting it right every time. J Antimicrob Chemother. (2021) 76:813–9. doi: 10.1093/jac/dkaa478, PMID: 33219669

[3] Bassetti M Vena A Labate L Giacobbe DR. Empirical antibiotic therapy for difficult-to-treat gram-negative infections: when, how, and how Long? Curr Opin Infect Dis. (2022) 35:568–74. doi: 10.1097/QCO.0000000000000884, PMID: 36206149

[4] Vena A Schenone M Corcione S Giannella M Pascale R Giacobbe DR. Impact of adequate empirical combination therapy on mortality in septic shock due to Pseudomonas Aeruginosa bloodstream infections: a multicentre retrospective cohort study. J Antimicrob Chemother. (2024) 79:2846–53. doi: 10.1093/jac/dkae296, PMID: 39224938

[5] Zilberberg MD Shorr AF Micek ST Vazquez-Guillamet C Kollef MH. Multi-drug resistance, inappropriate initial antibiotic therapy and mortality in gram-negative severe Sepsis and septic shock: a retrospective cohort study. Crit Care. (2014) 18:596. doi: 10.1186/s13054-014-0596-8, PMID: 25412897 PMC4264255

[6] Giacobbe DR Marelli C Cattardico G Fanelli C Signori A Di Meco G. Mortality in Kpc-producing Klebsiella Pneumoniae bloodstream infections: a changing landscape. J Antimicrob Chemother. (2023) 78:2505–14. doi: 10.1093/jac/dkad262, PMID: 37606528

[7] Giacobbe DR Signori A Tumbarello M Ungaro R Sarteschi G Furfaro E. Desirability of outcome ranking (door) for comparing diagnostic tools and early therapeutic choices in patients with suspected Candidemia. Eur J Clin Microbiol Infect Dis. (2019) 38:413–7. doi: 10.1007/s10096-018-3441-1, PMID: 30506332

[8] Maraolo AE Corcione S Grossi A Signori A Alicino C Hussein K. The impact of Carbapenem resistance on mortality in patients with *Klebsiella Pneumoniae* bloodstream infection: an individual patient data Meta-analysis of 1952 patients. Infect Dis Ther. (2021) 10:541–58. doi: 10.1007/s40121-021-00408-8, PMID: 33586088 PMC7954918

[9] Bonazzetti C Rocchi E Toschi A Derus NR Sala C Pascale R. Artificial intelligence model to predict resistances in gram-negative bloodstream infections. NPJ Digit Med. (2025) 8:319. doi: 10.1038/s41746-025-01696-x, PMID: 40442363 PMC12122886

[10] Gallardo-Pizarro A Teijon-Lumbreras C Monzo-Gallo P Aiello TF Chumbita M Peyrony O. Development and validation of a machine learning model for the prediction of bloodstream infections in patients with hematological malignancies and febrile neutropenia. Antibiotics (Basel). (2024) 14:13. doi: 10.3390/antibiotics14010013, PMID: 39858299 PMC11760484

[11] Giacobbe DR Guastavino S Razzetta A Marelli C Mora S Russo C. Deep learning for the early diagnosis of Candidemia. Infect Dis Ther. (2025) 14:1529–45. doi: 10.1007/s40121-025-01171-w, PMID: 40549343 PMC12270986

[12] Giacobbe DR Marelli C Guastavino S Signori A Mora S Rosso N. Artificial intelligence and prescription of antibiotic therapy: present and future. Expert Rev Anti-Infect Ther. (2024) 22:819–33. doi: 10.1080/14787210.2024.2386669, PMID: 39155449

[13] Meng Q Chen B Xu Y Zhang Q Ding R Ma Z. A machine learning model for early Candidemia prediction in the intensive care unit: clinical application. PLoS One. (2024) 19:e0309748. doi: 10.1371/journal.pone.0309748, PMID: 39250466 PMC11383240

[14] Ripoli A Sozio E Sbrana F Bertolino G Pallotto C Cardinali G. Personalized machine learning approach to predict Candidemia in medical wards. Infection. (2020) 48:749–59. doi: 10.1007/s15010-020-01488-3, PMID: 32740866

[15] Villani Junior A Freire MP Lazar Neto F Lage L Oliveira MS Abdala E. Prediction of bacterial and fungal bloodstream infections using machine learning in patients undergoing chemotherapy. Eur J Cancer. (2025) 223:115516. doi: 10.1016/j.ejca.2025.115516, PMID: 40382858

[16] Yuan S Xu S Lu X Chen X Wang Y Bao R. A privacy-preserving platform oriented medical healthcare and its application in identifying patients with Candidemia. Sci Rep. (2024) 14:15589. doi: 10.1038/s41598-024-66596-8, PMID: 38971879 PMC11227531

[17] Bassetti M Giacobbe DR Vena A Brink A. Challenges and research priorities to Progress the impact of antimicrobial stewardship. Drugs Context. (2019) 8:212600. doi: 10.7573/dic.212600, PMID: 31516534 PMC6726362

[18] Dyar OJ Huttner B Schouten J Pulcini C Esgap. What is antimicrobial stewardship? Clin Microbiol Infect. (2017) 23:793–8. doi: 10.1016/j.cmi.2017.08.026 28882725

[19] Giacobbe DR Marelli C La Manna B Padua D Malva A Guastavino S. Advantages and limitations of large language models for antibiotic prescribing and antimicrobial stewardship. NPJ Antimicrob Resist. (2025) 3:14. doi: 10.1038/s44259-025-00084-5, PMID: 40016394 PMC11868396

[20] Hibbard R Mendelson M Page SW Ferreira JP Pulcini C Paul MC. Antimicrobial stewardship: a definition with a one health perspective. NPJ Antimicrob Resist. (2024) 2:15. doi: 10.1038/s44259-024-00031-w, PMID: 39843968 PMC11721127

[21] Johnson MD Lewis RE Dodds Ashley ES Ostrosky-Zeichner L Zaoutis T Thompson GR. Core recommendations for antifungal stewardship: a statement of the mycoses study group education and research consortium. J Infect Dis. (2020) 222:S175–98. doi: 10.1093/infdis/jiaa394, PMID: 32756879 PMC7403757

[22] Miyazaki T Kohno S. Current recommendations and importance of antifungal stewardship for the Management of Invasive Candidiasis. Expert Rev Anti-Infect Ther. (2015) 13:1171–83. doi: 10.1586/14787210.2015.1058157 26089168

[23] Pulcini C. Antibiotic stewardship: update and perspectives. Clin Microbiol Infect. (2017) 23:791–2. doi: 10.1016/j.cmi.2017.08.020, PMID: 28882723

[24] Daikos GL Vryonis E Psichogiou M Tzouvelekis LS Liatis S Petrikkos P. Risk factors for bloodstream infection with *Klebsiella Pneumoniae* producing Vim-1 Metallo-Beta-lactamase. J Antimicrob Chemother. (2010) 65:784–8. doi: 10.1093/jac/dkq005, PMID: 20106862

[25] Giannella M Trecarichi EM De Rosa FG Del Bono V Bassetti M Lewis RE. Risk factors for Carbapenem-resistant *Klebsiella Pneumoniae* bloodstream infection among rectal carriers: a prospective observational multicentre study. Clin Microbiol Infect. (2014) 20:1357–62. doi: 10.1111/1469-0691.12747, PMID: 24980276

[26] Perez-Galera S Bravo-Ferrer JM Paniagua M Kostyanev T de Kraker MEA Feifel J. Risk factors for infections caused by Carbapenem-resistant Enterobacterales: an international matched case-control-control study (Eureca). EClinicalMedicine. (2023) 57:101871. doi: 10.1016/j.eclinm.2023.101871, PMID: 36895801 PMC9989660

[27] Tumbarello M Trecarichi EM Tumietto F Del Bono V De Rosa FG Bassetti M. Predictive models for identification of hospitalized patients harboring Kpc-producing *Klebsiella Pneumoniae*. Antimicrob Agents Chemother. (2014) 58:3514–20. doi: 10.1128/AAC.02373-13, PMID: 24733460 PMC4068482

[28] James G Witten D Hastie T Tibshirani R. Classification In: James G Witten D Hastie T Tibshirani R, editors. An introduction to statistical learning: With applications in R. New York, NY: Springer US (2021). 129–95.

[29] Calderon-Parra J Carretero-Henriquez MT Escudero G Suances-Martin E Murga de la Fuente M Gonzalez-Merino P. Current epidemiology, risk factors and influence on prognosis of multidrug resistance in Klebsiella Spp. bloodstream infection. Insights from a prospective cohort. Eur J Intern Med. (2025) 139:106369. doi: 10.1016/j.ejim.2025.05.034, PMID: 40506298

[30] Cao H Zhou S Wang X Xiao S Zhao S. Risk factors for multidrug-resistant and Carbapenem-resistant *Klebsiella Pneumoniae* bloodstream infections in Shanghai: a five-year retrospective cohort study. PLoS One. (2025) 20:e0324925. doi: 10.1371/journal.pone.0324925, PMID: 40403001 PMC12097643

[31] Cheng Y Cheng Q Zhang R Gao JY Li W Wang FK. Retrospective analysis of molecular characteristics, risk factors, and outcomes in Carbapenem-resistant *Klebsiella Pneumoniae* bloodstream infections. BMC Microbiol. (2024) 24:309. doi: 10.1186/s12866-024-03465-4, PMID: 39174950 PMC11340057

[32] Guan J Ren Y Dang X Gui Q Zhang W Lu Z. Predictive model for Carbapenem-resistant *Klebsiella Pneumoniae* bloodstream infection based on a nomogram: a retrospective study. BMC Res Notes. (2025) 18:265. doi: 10.1186/s13104-025-07325-w, PMID: 40598640 PMC12220204

[33] Kong H Liu Y Yang L Chen Q Li Y Hu Z. Seven-year change of prevalence, clinical risk factors, and mortality of patients with Carbapenem-resistant *Klebsiella Pneumoniae* bloodstream infection in a Chinese teaching hospital: a case-case-control study. Front Microbiol. (2025) 16:1531984. doi: 10.3389/fmicb.2025.1531984, PMID: 40177489 PMC11962001

[34] Simsek Bozok T Bozok T Sahinoglu MS Kaya H Horasan ES Kaya A. Bloodstream infections caused by carbapenem-resistant *Klebsiella pneumoniae*: analysis of risk factors, treatment responses and mortality. Infect Dis (Lond). (2025) 57:350–60. doi: 10.1080/23744235.2024.2436991 39661639

[35] Peiffer-Smadja N Rawson TM Ahmad R Buchard A Georgiou P Lescure FX. Machine learning for clinical decision support in infectious diseases: a narrative review of current applications. Clin Microbiol Infect. (2020) 26:584–95. doi: 10.1016/j.cmi.2019.09.009, PMID: 31539636

[36] Macukow B. Neural networks – state of art, brief history, basic models and architecture In: Saeed K, Homenda W, editors. Computer information systems and industrial management. Cham: Springer International Publishing (2016)

[37] Montesinos López OA Montesinos López A Crossa J. Overfitting, model tuning, and evaluation of prediction performance In: Montesinos López OA Montesinos López A Crossa J, editors. Multivariate statistical machine learning methods for genomic prediction. Cham: Springer International Publishing (2022). 109–39. 36103587

[38] Sarker IH. Deep learning: a comprehensive overview on techniques, taxonomy, applications and research directions. SN Computer Sci. (2021) 2:420. doi: 10.1007/s42979-021-00815-1, PMID: 34426802 PMC8372231

[39] Bradley AP. The use of the area under the roc curve in the evaluation of machine learning algorithms. Pattern Recogn. (1997) 30:1145–59. doi: 10.1016/S0031-3203(96)00142-2

[40] Marandi RZ Hertz FB Thomassen JQ Rasmussen SC Frikke-Schmidt R Frimodt-Moller N. Prediction of bloodstream infection using machine learning based primarily on biochemical data. Sci Rep. (2025) 15:17478. doi: 10.1038/s41598-025-01821-6, PMID: 40394073 PMC12092749

[41] Rahmani K Garikipati A Barnes G Hoffman J Calvert J Mao Q. Early prediction of central line associated bloodstream infection using machine learning. Am J Infect Control. (2022) 50:440–5. doi: 10.1016/j.ajic.2021.08.017, PMID: 34428529

[42] Cappello A Murgia Y Giacobbe DR Mora S Gazzarata R Rosso N. Automated extraction of standardized antibiotic resistance and prescription data from laboratory information systems and electronic health records: a narrative review. Front Antibiot. (2024) 3:1380380. doi: 10.3389/frabi.2024.1380380, PMID: 39816258 PMC11731964

[43] Weiskopf NG Hripcsak G Swaminathan S Weng C. Defining and measuring completeness of electronic health Records for Secondary use. J Biomed Inform. (2013) 46:830–6. doi: 10.1016/j.jbi.2013.06.010, PMID: 23820016 PMC3810243

[44] Guggilla V Kang M Bak MJ Tran SD Pawlowski A Nannapaneni P. Large language models outperform traditional structured data-based approaches in identifying immunosuppressed patients. medRxiv. (2025). doi: 10.1101/2025.01.16.25320564

[45] Mora S Giacobbe DR Bartalucci C Viglietti G Mikulska M Vena A. Towards the automatic calculation of the equal Candida score: extraction of Cvc-related information from Emrs of critically ill patients with Candidemia in intensive care units. J Biomed Inform. (2024) 156:104667. doi: 10.1016/j.jbi.2024.104667, PMID: 38848885

[46] Rudin C. Stop explaining black box machine learning models for high stakes decisions and use interpretable models instead. Nature Machine Intell. (2019) 1:206–15. doi: 10.1038/s42256-019-0048-x, PMID: 35603010 PMC9122117

[47] Giacobbe DR Marelli C Guastavino S Mora S Rosso N Signori A. Explainable and interpretable machine learning for antimicrobial stewardship: opportunities and challenges. Clin Ther. (2024) 46:474–80. doi: 10.1016/j.clinthera.2024.02.010, PMID: 38519371

[48] Ali S Akhlaq F Imran AS Kastrati Z Daudpota SM Moosa M. The enlightening role of explainable artificial intelligence in Medical & Healthcare Domains: a systematic literature review. Comput Biol Med. (2023) 166:107555. doi: 10.1016/j.compbiomed.2023.107555, PMID: 37806061

[49] Cavallaro M Moran E Collyer B McCarthy ND Green C Keeling MJ. Informing antimicrobial stewardship with explainable Ai. PLoS Digit Health. (2023) 2:e0000162. doi: 10.1371/journal.pdig.0000162, PMID: 36812617 PMC9931350

[50] Ghassemi M Oakden-Rayner L Beam AL. The false Hope of current approaches to explainable artificial intelligence in health care. The Lancet Digital Health. (2021) 3:e745–50. doi: 10.1016/S2589-7500(21)00208-9, PMID: 34711379

[51] Renftle M Trittenbach H Poznic M Heil R. What do algorithms explain? The issue of the goals and capabilities of explainable artificial intelligence (Xai). Humanit Soc Sci Commun. (2024) 11:760. doi: 10.1057/s41599-024-03277-x

[52] Giacobbe DR Bassetti M. The fading structural prominence of explanations in clinical studies. Int J Med Inform. (2025) 197:105835. doi: 10.1016/j.ijmedinf.2025.105835, PMID: 39951961

[53] Beauchamp TL Childress JF. Principles of biomedical ethics. 8th ed. Oxford: Oxford Publishing Press (2019).

[54] Yoo J Kim SH Hur S Ha J Huh K Cha WC. Candidemia risk prediction (Candetec) model for patients with malignancy: model development and validation in a single-center retrospective study. JMIR Med Inform. (2021) 9:e24651. doi: 10.2196/24651, PMID: 34309570 PMC8367162

[55] Yuan S Sun Y Xiao X Long Y He H. Using machine learning algorithms to predict Candidaemia in Icu patients with new-onset systemic inflammatory response syndrome. Front Med (Lausanne). (2021) 8:720926. doi: 10.3389/fmed.2021.720926, PMID: 34490306 PMC8416760

[56] Leon C Ruiz-Santana S Saavedra P Almirante B Nolla-Salas J Alvarez-Lerma F. A bedside scoring system ("Candida score") for early antifungal treatment in nonneutropenic critically ill patients with Candida colonization. Crit Care Med. (2006) 34:730–7. doi: 10.1097/01.CCM.0000202208.37364.7D, PMID: 16505659

[57] Leon C Ruiz-Santana S Saavedra P Galvan B Blanco A Castro C. Usefulness of the "Candida score" for discriminating between Candida colonization and invasive candidiasis in non-neutropenic critically ill patients: a prospective multicenter study. Crit Care Med. (2009) 37:1624–33. doi: 10.1097/CCM.0b013e31819daa14, PMID: 19325481

[58] Yang J Eyre DW Lu L Clifton DA. Interpretable machine learning-based decision support for prediction of antibiotic resistance for complicated urinary tract infections. NPJ Antimicrob Resistance. (2023) 1:14. doi: 10.1038/s44259-023-00015-2, PMID: 38686216 PMC11057209

[59] Vihta K-D Pritchard E Pouwels KB Hopkins S Guy RL Henderson K. Predicting future hospital antimicrobial resistance prevalence using machine learning. Commun Med. (2024) 4:197. doi: 10.1038/s43856-024-00606-8, PMID: 39390045 PMC11467333

[60] Hanscheid T Carrasco J Grobusch MP. Re: 'comparing large language models for antibiotic prescribing in different clinical scenarios' by De Vito et Al. Clin Microbiol Infect. (2025). doi: 10.1016/j.cmi.2025.08.006 40812548

